# Methane-to-chemicals: a pathway to decarbonization

**DOI:** 10.1093/nsr/nwad116

**Published:** 2023-04-25

**Authors:** Nikolai Nesterenko, Izabel C Medeiros-Costa, Edwin B Clatworthy, Hugo Cruchade, Stanislav V Konnov, Jean-Pierre Dath, Jean-Pierre Gilson, Svetlana Mintova

**Affiliations:** TotalEnergies One Tech Belgium, Zone Industrielle C, Seneffe 7181, Belgium; TotalEnergies One Tech Belgium, Zone Industrielle C, Seneffe 7181, Belgium; Laboratoire Catalyse et Spectrochimie (LCS), ENSICAEN, CNRS, Normandie Université, Caen 14050, France; Laboratoire Catalyse et Spectrochimie (LCS), ENSICAEN, CNRS, Normandie Université, Caen 14050, France; Laboratoire Catalyse et Spectrochimie (LCS), ENSICAEN, CNRS, Normandie Université, Caen 14050, France; TotalEnergies One Tech Belgium, Zone Industrielle C, Seneffe 7181, Belgium; Laboratoire Catalyse et Spectrochimie (LCS), ENSICAEN, CNRS, Normandie Université, Caen 14050, France; TotalEnergies One Tech Belgium, Zone Industrielle C, Seneffe 7181, Belgium

**Keywords:** methane, chemicals, pathways, decarbonization

## Abstract

The utilization of methane for chemical production, often considered as the future of petrochemistry, historically could not compete economically with conventional processes due to higher investment costs. Achieving sustainability and decarbonization of the downstream industry by integration with a methane-to-chemicals process may provide an opportunity to unlock the future for these technologies. Gas-to-chemicals is an efficient tool to boost the decarbonization potential of renewable energy. While the current implementation of carbon capture utilization and storage (CCUS) technologies is of great importance for industrial decarbonization, a shift to greener CO_2_-free processes and CO_2_ utilization from external sources for manufacturing valuable goods is highly preferred. This review outlines potential options for how a methane-to-chemicals process could support decarbonization of the downstream industry.

## Teaser

Methane-to-chemicals processes provide an opportunity to boost the decarbonization potential of renewable energy and to unlock novel technologies for creating sustainability value.

## SETTING THE SCENE

Addressing the issue of climate change is a global priority. At the Paris Climate Conference (COP21) in December 2015 an unprecedented legally binding global climate agreement was signed by 195 countries [1]. More remarkably, despite the COVID-19 pandemic, the year 2020 witnessed major international and national oil companies and governments commit to net zero carbon emission by 2050 [1]. Net zero emissions and carbon neutrality does not imply that fossil resources will disappear completely, but global greenhouse gas (GHG) emissions must decrease by 90%, relative to 2020, which will limit global warming to 1.5 degrees [2]. This is a tremendous challenge given that economic growth is forecasted to double in size by 2050 [3]. Different strategies based on divestment of polluting industries, decarbonization including carbon sinks, circularity, carbon capture and sequestration (CCUS technologies), carbon offsets, and clean energy solutions will be key to achieving carbon neutrality. The variety of renewable and clean energy sources will multiply, including solar, wind, biomass, geothermal, and many other emerging zero/low-carbon energy sources. However, these energy sources would not necessarily generate energy suitable for the consumer market at appropriate locations, and thus a transformation of renewable energy to a clean energy vector, including hydrogen, renewable natural gas, methanol, ammonia, or other types of sustainable fuels will be required. Despite significant investments in renewable energy production there will be a remaining shortfall of energy supply during the energy transition period. Thus, the most efficient utilization of renewable energy sources for enhancing decarbonization must be considered. An example is the production of chemicals while simultaneously extracting hydrogen from methane to be used as a clean energy vector. The energy content per carbon number in methane is higher than in chemical products, so there is the potential to extract the excess energy via low-carbon methane-to-chemicals processes. In these processes the fossil-based carbon will remain in the chemical products and the co-produced hydrogen will provide sustainability value in addition to significant economic revenue, unlocking the future of these technologies.

Methane-to-chemicals technologies provide an opportunity to efficiently use this valuable resource, multiplying the impact of renewable energy on decarbonization. A shift to greener CO_2_-free technologies is often linked with a source of hydrogen, which is an irreplaceable raw material for many products and could be employed as a clean energy vector. The amount of electrical energy for H_2_ production from methane is significantly lower in comparison with state-of-the art water electrolysis (*vide infra*). Both technologies produce CO_2_-free renewable H_2_. This latter aspect will attract significant interest toward CO_2_-free electrified processes due to on-going market developments for renewable hydrogen production and a fast-growing availability of renewable energy. In addition, electrified reactors are not the only way to decarbonize energy, but also an efficient technological solution to produce chemicals from methane with a superior yield relative to thermochemical routes. Methane is the second most important GHG after CO_2_, despite its shorter mean atmospheric lifetime, due to its higher global warming potential and accounts for ∼16% of global anthropogenic emissions [4,5]. The captured methane typically has no local use and is flared [6].

**Figure 1.**
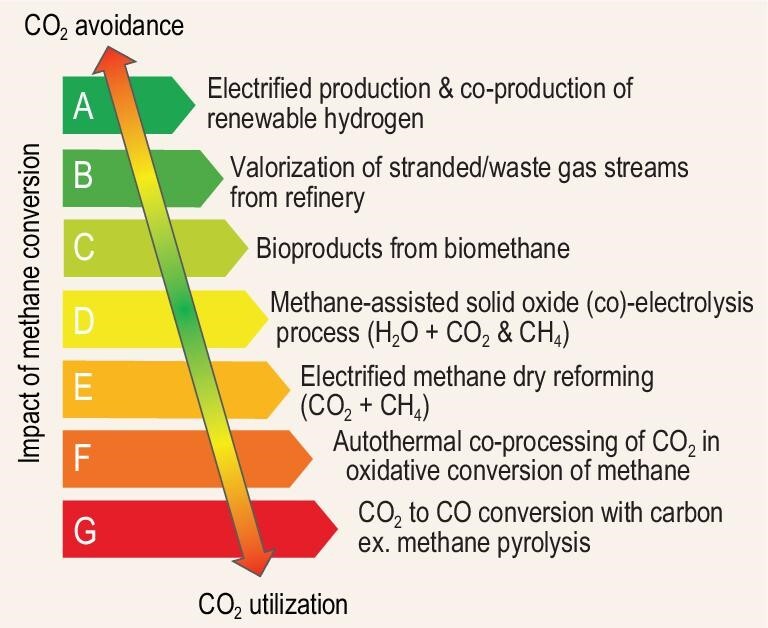
The impact of different pathways for methane conversion on decarbonization represented by the tendency from CO_2_ avoidance to CO_2_ utilization in the scale from A (the most efficient: electrified production and renewable hydrogen co-production), B (valorization of waste gas streams), C (bioproducts production from biomethane), D (methane assisted solid oxide electrolysis), E (electrified methane dry reforming), F (co-processing of CO_2_ in oxidative conversion of methane) to G (the less desired: methane pyrolysis).

Processes utilizing CO_2_ for the production of goods and fuels is an important element of the circular economy. Conversion of CO_2_ to CO without any external supply of H_2_, such as in autothermal co-processing of natural gas with CO_2_, offers substantial potential to avoid GHG emissions from the chemical and refining industry. This is made possible by providing viable options for CO_2_ circularity while employing existing local industry. Methane-to-chemicals also contributes to the decarbonization of refinery and petrochemical complexes by avoiding CO_2_ emissions from the flaring of off-gases, providing solutions for the valorization of those emission streams and by displacing traditional H_2_ production routes of grey/blue hydrogen with a more sustainable one (Fig. 1). Thanks to the utilization of the energy difference between the feedstock and products, these solutions are more energy efficient and less expensive than any of the on-purpose CO_2_ utilization facilities. There are excellent prospects to successfully address the ‘Methane Challenge’ with the current developments in the preparation of bi-functional stable materials for autothermal processes and cold-wall electrified reactors. These technologies will eventually become cheaper and become both an important source of chemicals and a driver of decarbonization. The current focus is on searching for the most efficient option to valorize the existing knowledge and achievements in catalysis for the electrified conversion of natural gas.

Natural gas, the cleanest burning fossil fuel, is a highly efficient form of energy and can reduce, in the short term, the overall GHG emission intensity of the energy sector by displacing coal [7,8]. This means that, for the same amount of delivered energy, methane emits roughly three times less CO_2_, less than a tenth of sulfur oxides, a quarter of nitrogen oxides, and essentially no particulate matter or heavy metals compared to coal (Figure S1) [9]. Natural gas is easier to purify upstream compared to oil and the products delivered to customers are almost completely free of impurities (sulfur, nitrogen, metals). A cubic meter of methane contains the same amount of energy as 1.1 L of gasoline and 1.8 L of bioethanol, and could be used to generate 5.6 kWh of electricity [9]. If methane is used as a feedstock for chemical transformation, it could produce approximately 450 g of plastics or 180 g of hydrogen and 536 kg of carbon product [10–12]. At present, methane conversion is of particular importance due to its high hydrogen content and availability including from bio-sources [13,14]. Besides its use in power generation, methane is currently the primary source for hydrogen production. Steam methane reforming (SMR) using natural gas is the workhorse for hydrogen production in the ammonia and methanol industries and refineries. Natural gas accounts for ∼60% of the dedicated global production of hydrogen [15]. The downside of hydrogen production by SMR is the significant emission of CO_2_ of about 11.9 ton CO_2_ equivalent per ton of H_2_, which needs to be captured and stored [16].

## IMPACT OF DIFFERENT METHANE CONVERSION PATHWAYS ON DECARBONIZATION

At present it is likely that there will be no requirement of an additional process for the on-purpose production of fossil-based olefins or aromatics. However, the implementation of a process to help the downstream industry with CO_2_ utilization and provide a supply of low-carbon hydrogen is highly desirable. This represents a paradigm shift where sustainability is the main product and fossil-based chemicals are a carbon sink with significant market value. The sustainability value will be highest for methane feedstock and will also offer one of the most efficient utilizations of renewable electricity. Currently, few reports pay attention to the hidden potential of natural gas conversion to chemicals in achieving net zero objectives. In order to underpin this statement, one can define an efficiency metric with the key sustainability drivers for a gas-to-chemicals process including the utilization of water resources, synergy with traditional petrochemistry, valorization of waste streams, co-production of low carbon/renewable H_2_, electrification of assets, and CO_2_ sink opportunities.

There are several different approaches for utilizing the excess energy from methane conversion for decarbonization: autothermal conversion, electrification with either the production of hydrogen or co-processing with CO_2_, biomethane feedstock, or the utilization of soft oxidants. These approaches provide different conversion routes with a different impact on CO_2_ reduction which is presented in Fig. 1.

### Autothermal conversion

To date, the main mature processes for valorizing methane-rich streams are ‘Methanol-To-Olefins’ (MTO) [17–20] and Fischer-Tropsch (FT) [21]. By using synthesized gas as an intermediate a wide range of products including light olefins and aromatics can be accessed. However, the large amount of intermediate steps, a significantly higher capital investment [21,22], and a significant CO_2_ footprint compared to conventional petrochemistry processes represent significant drawbacks. Therefore these processes have found commercial applications in only a few particular regions of the world [23,24]. Recently, both MTO and FT have received significant attention due to their potential for syngas valorization from CO_2_-enriched feedstocks in a variety of autothermal processes involving methane [25–27]. The principle of autothermal conversion is based on the utilization of excess energy from methane transformation to drive a second endothermic reaction, e.g. CO_2_ utilization. In these combined processes the excess energy is directly used by the second process or is extracted as hydrogen. The autothermal conversion of natural gas offers substantial potential for the decarbonization of the chemical and refining industry by avoiding GHG emissions and integrating CO_2_ circularity within existing assets (F, Fig. 1).

Initially, auto-thermal reforming (ATR) has been proposed for low-carbon methane-to-syngas processes, as an alternative to SMR with post-combustion capture, for hydrogen production with low CO_2_ emissions [28]. In the ATR concept, a portion of the natural gas feed is partially oxidized to generate the heat required to carry out the endothermic reforming reactions. Combining a partial oxidation reactor in series with a SMR reactor resulted in syngas with the required H_2_:CO ratio for downstream upgrading, e.g. methanol or Fischer-Tropsch synthesis. ATR is a process where methane is converted into syngas by the addition of oxygen and steam. The main desired reactions are as follows:


\begin{eqnarray*} && {\rm{Partial\, oxidation{:}}}\ {{\rm{CH}}}_{\rm{4}}+ {^1}/{_2}{{\rm{O}}}_{\rm{2}} \to {\rm{CO + 2}} {{\rm{H}}}_{\rm{2}}\\ && {\rm{ \Delta H = }} - {\rm{36\ kJ / mol}} \end{eqnarray*}

\begin{eqnarray*} && {\rm{Steam{-} Methane{-}Reforming}}\, ({{\rm{SMR}}}){:}\\ && {\rm{C}}{{\rm{H}}}_{\rm{4}}+ {{\rm{H}}}_{\rm{2}}{\rm{O}} \to {\rm{CO + 3 }}{{\rm{H}}}_{\rm{2}}\\ && \Delta {\rm{H = + 206\ kJ / mol}} \end{eqnarray*}

Regarding hydrogen production, the ATR technology facilitates CO_2_ capture (up to 95%) due to the significantly lower concentration of CO_2_ in flue gases.

Recently, a ‘Super-dry’ CH_4_ reforming reaction for enhanced CO production from CH_4_ and CO_2_ was proposed [26]:


\begin{eqnarray*} && {\rm `Super-dry\,reforming':}\\ && {{\rm{CO}}_{\rm{2}} + {^1}/{_3}}{\rm{C}}{{\rm{H}}}_{\rm{4}} \to {\scriptstyle 4} /{\scriptstyle 3}{\rm{CO }}+{\scriptstyle 2} /{\scriptstyle 3}{{\rm{H}}}_{\rm{2}}{\rm{O }}\\ && \Delta {\rm{H = + 109}}{\rm{.9\ kJ / mol}} \end{eqnarray*}

\begin{eqnarray*} && {\rm{Dry\ Reforming{:}}}\\ && {\rm{C}}{{\rm{O}}}_{\rm{2}}+{\rm{ C}}{{\rm{H}}}_{\rm{4}} \to {\rm{2CO + 2}}{{\rm{H}}}_{{\rm{2\ }}}{\rm}\\ && \Delta {\rm{H = + 247\ kJ / mol}} \end{eqnarray*}

The idea of ‘Super-dry’ reforming is based on utilization of the hydrogen produced by a dry reforming process *in situ* to perform the reverse water-gas-shift (RWGS) reaction. The catalysts comprise of a combination of a classic reforming catalyst (e.g. Ni/MgAl_2_O_4_), a solid oxygen carrier (Fe_2_O_3_/MgAl_2_O_4_), and a CO_2_ sorbent (e.g. CaO/Al_2_O_3_) [26]. The autothermal coupling of these three different processes resulted in higher CO_2_ utilization per mol of converted methane compared with the conventional dry reforming process. The advantage of the ‘Super-dry’ reforming process is a lower energy requirement to transform a mole of CO_2_ to CO (2.5 times lower), simplifying the reactor design and decreasing the energy used for product purification. Recent work has demonstrated this process can be enhanced further using up to 2.9 mol of CO_2_ per mol of CH_4_ [29].

In order to further decarbonize the production of syngas, several studies have focused on the tri-reforming process as a novel autothermal technology for flue gas treatment whereby the exhausts are directly used to generate a sustainable synthesis gas [27]. Typically, a flue gas contains CO_2_, water, and oxygen. The upgrading of such a composition by the addition of methane leads to the synergetic combination of CO_2_ reforming, steam reforming and partial oxidation of methane in a single reactor. This can produce syngas with a H_2_:CO ratio of ∼2.0 which is necessary for methanol and gas-to-liquids fuel production. In addition, it will eliminate carbon formation which is a serious problem in the CO_2_ dry reforming of methane. The main advantage is that both greenhouse gases, CO_2_ and CH_4_, can be converted into a useful product.

The tri-reforming process can be also performed in an electrified reactor with *in situ* generation of oxygen from CO_2_/H_2_O co-electrolysis in a solid oxide electrolysis cell (SOEC):


\begin{eqnarray*} {{\rm{H}}}_{\rm{2}}{\rm{O}} + {\rm{ }}2{e}^ - \to {\rm{ }}{{\rm{H}}}_{\rm{2}} +{{\rm{O}}}^{{\rm{2}} - } \left( {{\rm{cathodic\ half\ reaction}}} \right)\end{eqnarray*}

\begin{eqnarray*} \\{\rm{C}}{{\rm{O}}}_2 + 2{e}^ - \to {\rm{CO}} + {\rm{ }}{{\rm{O}}}^{2 - } \left( {{\rm{cathodic\ half\ reaction}}} \right)\end{eqnarray*}

\begin{eqnarray*} {{\rm{O}}}^{2 - } \to {^1}/{_2} {{\rm{O}}}_2 + {\rm{ }}2{e}^ - \left( {{\rm{anodic\ half\ reaction}}} \right) \end{eqnarray*}

\begin{eqnarray*} \\{\rm{C}}{{\rm{H}}}_4 + {\rm{ }}{{\rm{O}}}^{2 - } \to {\rm{ CO}} + 2{{\rm{H}}}_2 + 2{e}^ -\\ \left( {{\rm{anodic\ half\ reaction}}} \right)\end{eqnarray*}

Oxygen anions are transferred under the drive of electric load from the cathode to anode to produce O_2_ that could react with CH_4_ to produce more CO, syngas, or ethylene via the oxidative coupling of methane (OCM) [30]. The resulting mixture from both sides could be subsequently used for upgrading the feedstock of the downstream MeOH synthesis or Fischer-Tropsh processes (D, Fig. 1). The transformation of methane on the anode reduces the open-circuit voltage (reduced electricity consumption) thus improving the economic competitiveness of solid oxide co-electrolysis technology for syngas production thanks to the production of useful products from the methane [31].

Further examples of an autothermal process include the combination of the exothermic oxidative coupling and the endothermic steam reforming of methane for simultaneous production of ethylene and synthesis gas. A feasibility study was performed on a dual functional catalyst in a packed bed reactor equipped with a porous membrane for distributive oxygen feeding. The intraparticle heat-sink significantly reduced the total heat reaction and temperature gradients in the reactor, eliminating the need for expensive conventional cooling [32].

Another example is the development of the first commercially viable methane-to-ethylene process by exploiting the exothermicity of OCM to initiate the cracking of ethane to ethylene [33,34]. The first catalytic reaction zone was heated adiabatically to the necessary temperature to perform pyrolytic conversion of ethane to ethylene in the non-catalytic second zone. The additional ethane was injected into the second zone to consume the heat generated from the OCM process [33,34].

Conversion of CO_2_/C to 2CO (reverse Boudouard reaction, catalyzed by metal carbonates) can be an alternative quench reaction to absorb heat and produce pure CO as a feedstock for valorization in chemical production. This has an advantage over processes which result in various product mixtures of CO and H_2_ from methane. On the one hand SMR requires CO_2_ capture and sequestration, while on the other CO_2_ conversion from an external source reduces the amount of hydrogen to transform it into chemicals. Furthermore, this transformation offers several other opportunities including the utilization of carbon ex-methane pyrolysis (G, Fig. 1), and the direct employment of metal carbonates produced from CO_2_ sequestration as a feedstock without preliminary CO_2_ production. The benefit is the lower decomposition temperature of the carbonate, initially used to capture CO_2_, resulting in CO formation [35]:


\begin{eqnarray*} {\rm{C}}{{\rm{O}}}_3^{2 - } + {\rm{C}} \to {{\rm{O}}}^{2 - } + 2{\rm{CO}}. \end{eqnarray*}

The co-produced CO could be valorized in gas-to-chemicals processes while facilitating capture and utilization of the CO_2_ produced by other industries into the product pool. Recently a novel approach for carbon nanotube production from a mixture of CH_4_ and CO_2_ has been developed [36]. Carbon is a well-known by-product of the dry reforming process, but converting a mixture of CO_2_ and CH_4_ into a valuable carbon material is considered a promising carbon capture and sequestration technique. A novel technology known as CARGEN (CARbon GENerator) is using two reactors that separately convert the CO_2_ into multi-walled carbon nanotubes (MWCNTs) and syngas. The CARGEN reports at least a 50% reduction in energy with at least 65% CO_2_ conversion to carbon compared to the dry reforming process [36].

The development of autothermal conversion processes can facilitate the efficient use of energy from methane transformation for the utilization of CO_2_ and water splitting. In this context, the syngas conversion technology is of great importance and the concept of the CO/CO_2_ refinery is a part of the Horizon Europe program [37]. The CO/CO_2_ could be recycled in the refinery by a newly developed hydroformylation reaction, Fischer-Tropsch, or MeOH-type intermediate. The existing hydrotreatment unit may partially handle this type of chemistry if the appropriate catalyst can be developed and the reaction conditions adapted accordingly. It is important to note that the conversion of CO_2_ to CO has significant decarbonization value for the refining and petrochemical industry and is highly advantageous if the transformation can occur without requiring the isolation of hydrogen, such as directly with hydrocarbons (hydrocarbons + CO_2_), electrochemically, or with alternative reductive agents. Consequently, the value from the development of a new methane conversion process would come not only from valorization of the products but also from the decarbonization of assets.

### Electrification

Methane-to-chemicals offers a very efficient way to utilize, and to multiply the effect of, renewable energy on decarbonization. In addition, electrified reactors provide the possibility of employing conditions unattainable in a conventional thermochemical reactor. The latter will also result in the differentiation of those technologies in terms of higher selectivity and a simpler production slate. Thanks to better heat transfer, the reactor of an electrified reformer could be potentially ∼100 times smaller in comparison to a thermochemical one [38].

It is important to recognize that the electrification of a traditional petrochemical process, e.g. a steam cracker process, would be in direct competition with the state-of-the art thermochemical technology as well as many potentially cheaper alternative decarbonization options such as the optimization of energy consumption, electrification of compressors, cleaner fuel (H_2_, NH_3_, hythane), and CO_2_ capturing technologies. Most of the literature data on CO_2_ conversion to chemicals reports the results at very low current densities, in the range of 0.02–0.2 A·cm^−2^ [39–41]. An optimal balance between the maximization of selectivity, Faradaic efficiency, and productivity is necessary. The halogen (Hal) routes for methane activation utilizing Hal PEM electrolysis, have demonstrated Faradaic efficiencies and current densities in the range of 70%–80% and 0.9–1.6 A·cm^−2^, respectively [42]. In general, the electrified thermochemical reactor showed an efficiency above 90% and often higher than 95% [43]. Based on the current state of development, the production of hydrogen from methane via an electrified process followed by utilization of the CO_2_-free hydrogen for CO_2_ transformation seems to be one of the most efficient solutions. The electrification of a methane conversion process is the critical component for ensuring technological and economic feasibility. Considering strong competition for the best use of renewable electricity, electrified methane conversion processes are well positioned to emerge as frontrunners and show promising potential to compete globally with other decarbonization options.

Methane is the richest hydrocarbon in hydrogen content and its electrified conversion is often accompanied by CO_2_-free hydrogen co-production. This is an important economic driver which provides high value for the decarbonization of existing refining and petrochemical assets (A, Fig. 1). This will contribute to avoiding additional investment costs, higher energy consumption due to the energy for CO_2_ capture, as well as the requirement of access to the infrastructure and logistics for CO_2_ storage. Access to CO_2_ storage infrastructure is a fundamental problem for many existing refineries, especially in remote locations. In many cases the overall costs of switching to CO_2_-free hydrogen for a refinery utilizing grey hydrogen from SMR would be lower than investing in CO_2_ capture and storage infrastructure. In addition, CO_2_-free hydrogen can also be employed on-site for the conversion of CO_2_ to valuable products, e.g. methanol [44–49], or acetic acid [50].

Electrification of dry reforming is another attractive alternative [51]. This reaction is highly endothermic requiring exceptionally high temperatures to attain the high conversion of reactants to produce H_2_ and CO [52]. However, due to the presence of multiple thermodynamic equilibria the reaction environment is significantly diverse resulting in various side reactions depending on the operating conditions. Several plants for CO_2_-rich steam reforming are currently in operation [53]. The catalysts are based on Ni and Ru on a basic support, often doped with Cu, while the engineering of the catalyst support also plays an important role [52,54,55]. However, in the absence of steam the catalysts described above suffer from deactivation. The amount of water (CH_4_:H_2_O ratio) can be reduced for a dry reforming process in comparison to a state-of-the art SMR, but the presence of some steam would still be required for a conventional multi-tubular reactor design. In that context, the reaction may receive a second life thanks to the ongoing trends in electrification where more options for reactor design are feasible (E, Fig. 1).

### Biomethane feedstock

Another source of methane that could become a significant point of focus is the fast-growing production of biomethane from biogas [56]. The proper use of biomethane allows for a significant reduction in the carbon footprint of the energy sector while producing drop-in bioproducts (C, Fig. 1). There is already an established policy which contributes to the rapid development of biogas use [57,58]. Biomethane could be one of the cost-competitive sources for biofuel production, including bio-aviation fuel. In addition, biomethane is produced mainly from non-edible and non-nutritional feedstocks and remains a concentrated source of renewable energy. This has many advantages relative to wet biomass in terms of investment, purification and logistics. Conversion to fuels or chemicals by electrified methane pyrolysis could be an interesting valorization route for biogas to substitute low efficiency combined heat and power (CHP) plants in the future. In addition, the second major component of biogas, biogenic CO_2_, may also be considered as valuable as biomethane in the context of E-fuel production. Despite a relatively low production volume the valorization of biogas will play an important role in the implementation of a decentralized industry. This is key to securing development of the amount of renewable energy sufficient for what will be necessary to achieve living standards with zero-emissions.

### Utilization of soft oxidants

One of the recent emerging topics is the utilization of soft oxidants in the direct and indirect conversion of methane. In the oxygen-mediated direct conversion routes, the selectivity of a typical OCM process is significantly restricted by the over-oxidation to CO_x_ products and by a significant selectivity to ethane in the C_2_ fraction [59–62]. Other gaseous reagents such as N_2_O and sulfur have been investigated to a far lesser extent as milder alternative oxidants to replace O_2_. As such, N_2_O can also act as the oxidant for the OCM to form C_2_ products from the ethane-forming reaction. The enthalpy for the same coupling reaction is significantly lower in comparison with O_2_ [63]. Utilizing N_2_O as an oxidant leads to enhanced C_2_-selectivity in the OCM due to its relatively mild oxidizing power compared to O_2_ [63]. In particular, N_2_O can only provide a monoatomic oxygen species, such as O^−^. Dioxygen, in contrast, can form peroxy-species under OCM reaction conditions that are precursors for CO_2_ [64]. It was observed that with N_2_O, with limited-to-no competing diatomic or gas phase O_2_ formation, a very high C_2_ selectivity might be achieved at a high level of conversion. Additionally, the range of possible oxidant:CH_4_ ratios is far larger when N_2_O is used as there is no expected risk of explosion when compared to the use of O_2_ [65]. However, the costs of N_2_O synthesis and regeneration cannot justify its utilization for methane activation. In this context, utilization of sulfur is more attractive due to the state-of-the art Claus unit allowing the recovery of sulfur from H_2_S. S-mediated oxidative coupling (SOCM) over a sulfided Fe_3_O_4_ demonstrated stable conversion at 800°C and becomes more thermodynamically favorable at higher temperatures [66]. In contrast to the conventional OCM, the SOCM does not produce any ethane while ethylene is the main product in the C_2_-fraction. However, up to now, the C_2_ selectivity obtained via SOCM has been limited up to 18%. A significant amount of CS_2_ is co-produced which requires valorization [65].

**Figure 2.**
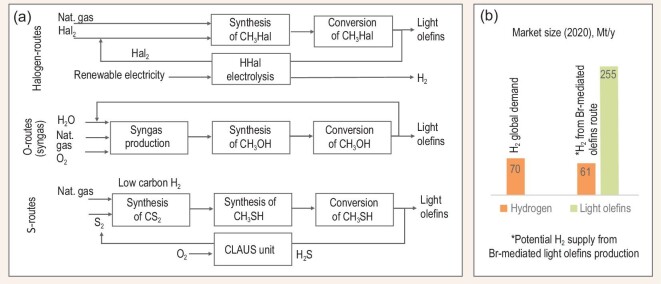
(a) Halogen-, oxygen-, and sulfur-mediated indirect routes for light olefins synthesis, and (b) global hydrogen demand and the H_2_-co-production potential for 255 Mt/y light olefins via halogenation pathway *vs* naphtha steam cracker [15,73].

Regarding the indirect routes, the halogen-mediated conversion is well known for the manufacturing of various important chemicals from methane (Fig. 2a). Historically, halogen chemistry played a central role at an industrial scale [67]. Utilization of a halogen allows the activation of methane at relatively mild conditions (350–450°C), leading to intermediate compounds which could be readily converted to valuable commodities. The advantage of the halogen route is that the methyl halides are obtained by a direct reaction between methane and the halogen without any intermediates, similar to syngas in case of the O-activation pathway. However, the downside of halogenation is the requirement to recover the halogen from the corresponding HHal (Hal representing a halogen atom). This is not necessary in the O-route because the process consumes oxygen and rejects water. Historically, recycling of the halogen was the economical showstopper of the technology. This problem could be partially mitigated via the oxyhalogenation pathway; however, a significant amount of CO_2_ and H_2_O are co-produced which reduces carbon efficiency and may complicate the selection of construction materials due to a higher risk of corrosion. Unexpectedly, an elegant solution for halogen recycling was found because of the increasing interest for green hydrogen production and a possibility to produce hydrogen from HHal by electrolysis requiring significantly lower energy expenditure relative to state-of-the-art water electrolysis.

Regarding the selection of the halogen, the reactivity weakens in the order of F > Cl > Br > I, with the increasing bond length between Hal − Hal; iodine (I_2_) is not sufficiently active to activate light paraffins [68]. In contrast, bromine is still sufficiently active to react directly with methane while showing the weakest Hal-Hal bond resulting in the lowest energy in Br-recovery and reuse. From this, Br_2_ is the optimal halogen for the transformation of methane to valuable products with the co-production of hydrogen.

Catalytic coupling of methyl halides occurs in the same way as in the conversion of methanol to olefins; zeolite materials (mostly CHA, MFI) are typically used as catalysts in these transformations [69–72]. By optimizing the catalyst composition, the yield of target chemicals from CH_3_Hal could be the same as from the commercial methanol conversion technology. Interestingly, the transformation of methyl halides is significantly less exothermic in comparison to MeOH which makes the reactor design significantly simpler. Due to the lower energy for Br-recovery from HBr, the Br-mediated processes are particularly interesting in view of chemicals synthesis from methane while co-producing hydrogen. The electrolysis of HBr requires roughly two times less energy than water electrolysis and may compensate the costs of halogen recovery. If the electricity for HBr electrolysis could originate form a renewable source, the CO_2_-free hydrogen produced in the process could be potentially certified as green. If the entire global demand for light olefins (255 Mt/y) was realized by the Br-mediated technology, the amount of co-produced green hydrogen would satisfy more than 85% of the global world hydrogen demand (Fig. 2b) [15,73]. It is interesting to note that the halogenation route allows for the generation of hydrogen almost 20 times higher in comparison with the state-of-the art naphtha steam cracker thus making the technology very promising for petrochemistry in the future.

A Br-mediated technology to transform methane to aromatics was recently reported by Sulzer showing promising carbon efficiency and interesting market potential [74].

In the same way as is the case for MeOH, synthesis of methyl mercaptan (CH_3_SH) cannot be performed directly from methane and requires an intermediate step [75]. However, in contrast to the O-routes (SMR), thio-reforming requires a much higher temperature due to the recombination of S_2_ with hydrogen. The most suitable intermediate in the S-route for the synthesis of methyl mercaptan will be CS_2_; the synthesis of CS_2_ is commercially performed via a reaction with elemental sulfur at relatively mild conditions in the temperature range of 550–600°C [76]. However, in the synthesis of CS_2_, hydrogen is not formed and the synthesis of CH_3_SH requires an external supply of hydrogen, a key disadvantage of this route. The produced CH_3_SH can be converted to ethylene and propylene in a similar manner as CH_3_OH over CHA zeolite and to a mixture of alkanes and aromatics on HZSM-5 [77,78]. The co-produced H_2_S can be sent to a Claus unit to be transformed back to elemental sulfur.

A comparison of the O-, S-, and Hal-mediated indirect routes to transform methane shows:

- The S-mediated route is the least advantageous due to the two step syntheses of CH_3_SH from methane (via the CS_2_ intermediate) with a requirement for an external hydrogen supply and the highest rate of recycling of the activation agent, i.e. the elemental sulfur.
- The O-route is well established but suffers from a significant capital intensity for CH_3_OH synthesis. The additional advantages of this technology may come from the CO_2_-utilizing route for syngas generation.
- The Br-route is the most promising due to the co-production of green hydrogen and one-step synthesis of CH_3_Br. This technology may produce a significant contribution to achieving the net zero objective and will emerge in the coming years.

## OPPORTUNITIES FOR GAS CONVERSION FROM TRANSFORMATION OF DOWNSTREAM INDUSTRY

The downstream industry emits more than 1000 Mt of CO_2_ per year; a problem that the sector will not be able to solve by itself [79]. Environmental sustainability will be a priority for refiners; finding their own unique decarbonization solutions and yet remain economically viable. Carbon capture and storage infrastructure is under development to mitigate this issue, but an increasing number of reports show that this will only delay the transition [80–82]. In that context, close integration of different industry sectors is required, and gas-to-chemicals processes are getting a unique opportunity to become a decarbonization engine for the downstream. Undoubtedly, the utilization of renewable energy offers unlimited potential for the decarbonization of the downstream industry. Large-scale applications of using renewable energy, such as ‘Liquid Sunshine’ solar fuels production, have demonstrated how renewable energy, water electrolysis, and CO_2_ hydrogenation can be integrated to produce MeOH on the thousands-ton scale [83]. However, despite significant investment in renewable energy production this resource will be remain in deficit during the energy transition period and the effect from its utilization needs to be levered.

**Figure 3.**
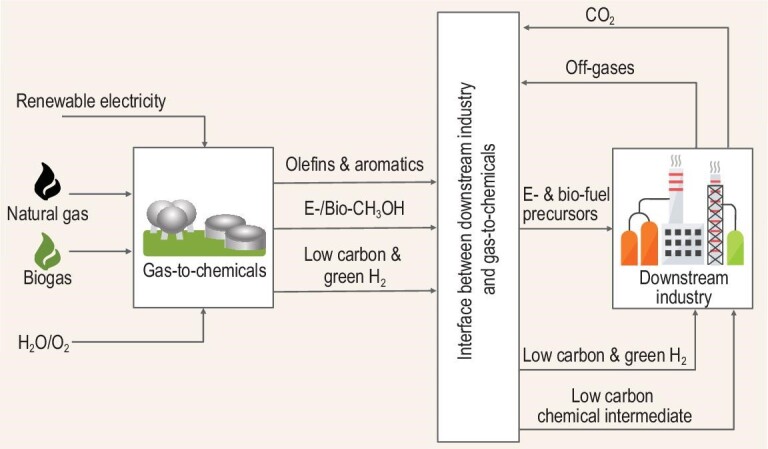
The role of gas conversion processes in decarbonization of the downstream industry: renewable electricity used to power gas-to-chemicals processes employing different methane sources (natural gas, biogas) to produce olefins, aromatics, e- and bio-methanol, and low carbon and green hydrogen. The interface between gas-to-chemicals and the downstream industry provides opportunities for utilization of CO_2_ and off-gases from the downstream industry for the production of e- and bio-fuels precursors, low carbon and green hydrogen, and low carbon chemical intermediates.

The upcoming trends in electrification of downstream processes together with the emerging expansion in petrochemicals (crude-to-chemicals) results in a lighter product slate in comparison with the traditional refinery, and in a necessity to valorize many methane-containing streams (off-gases). The Low-Carbon Emitting Technologies (LCET) initiative, led by the Chemistry and Advanced Material Governors Community at the World Economic Forum, set the objective to accelerate the development and upscaling of ‘electrified technologies’ and ‘alternative hydrogen production’. These ambitions to reduce GHG emissions via the electrification of the chemical industry could only be achieved if the on-purpose valorization of methane rich streams takes place and the off-gases will not be sent to flare or to fire heaters for energy production. Those emission mitigations may be achieved by utilization of the fuel gas in a different way, for instance, to produce useful products and clean energy vectors. It is well known that the most efficient way to reduce CO_2_ emissions is to not make them in the first place; achievable by increasing the supply for renewable energy. The latest of these will result in a significant avoidance of CO_2_ emissions from the flaring of those gases, as well as additional room for a CO_2_ sink from other assets (Fig. 3). However, it is still a matter of debate, for example, the Center for International Environmental Law claims that relying on the developments in carbon capture and storage technologies further delays the transformation of industry by giving too much credit to those technologies [82]. In addition, the methane emissions topic was addressed at the COP26 Summit and the methane emissions strategy will be a part of the EU Green Deal vision [84]. In order to mitigate the issue of methane emissions, decentralized methane monetization solutions should be urgently developed (B, Fig. 1). The global warming potential of methane is ∼28 times higher than that of CO_2_, therefore flaring is often used to mitigate the emission problem [4,5]. By transforming 1 ton of methane to 2.75 ton of CO_2_ in a flare, the impact from the direct methane emissions is roughly reduced ten times. In contrast, a methane-to-chemicals process potentially offers solutions to reduce the direct emissions of methane to nearly zero while contributing to the decarbonization of refinery and petrochemical complexes by avoiding and utilizing CO_2_ from flaring of off-gases. If renewable electricity is available, and assuming the current CO_2_ costs at 50$/t [85], the cumulative benefits from decarbonization alone via methane upgrading could provide between $137–183 per ton of methane as additional benefits. This is a result of 2.75 ton of CO_2_ avoidance from flaring of a ton of methane according to the first equation below resulting in $137 per ton of direct benefits. In addition, instead of producing CO_2_ in a flare, the same ton of methane could be transformed to about 125 kg of CO_2_-free hydrogen (see the second equation below), which may convert 917 kg of CO_2_ to MeOH (see the third equation below) [86]. The CO_2_ utilization would bring approximately an additional $46 per ton of methane.


\begin{eqnarray*} && {\rm{Combustion/Flaring{:} }}\\ && {{\rm{CH}}}_4 + {\rm{ }}2{{\rm{O}}}_2 \to {\rm{C}}{{\rm{O}}}_2 + {\rm{ }}2{{\rm{H}}}_2{\rm{O}} \end{eqnarray*}

\begin{eqnarray*}\\ \hbox{Methane-to-chemicals:}\ {{\rm{CH}}}_4 \to -{\rm{C}}{{\rm{H}}}_{{2}^ - } + {{\rm{H}}}_2\end{eqnarray*}

\begin{eqnarray*}\\ {\rm{C}}{{\rm{O}}}_2\ {\rm{ utilization{:} }}\ 3{{\rm{H}}}_2 + {\rm{C}}{{\rm{O}}}_2 \to {\rm{C}}{{\rm{H}}}_3{\rm{OH}} + {{\rm{H}}}_2{\rm{O}}\end{eqnarray*}

This value could more than double in the coming years with the growth in the price of CO_2_. Eventually, the costs of off-gas methane-containing streams may even become negative in the future and the bonuses from decarbonization will become comparable with the margins which could be obtained from chemical production. The figures will certainly keep growing in the near future.

## HYDROGEN AS A VECTOR FOR DECARBONIZATION

According to the Hydrogen Council [87], 18 governments whose economies account for more than 70% of global GDP, have developed hydrogen national strategies and issued their roadmaps toward a hydrogen economy by 2030. The number of countries with polices that directly support investment in hydrogen technologies keeps growing together with the number of targeted applications. In 2020, the global consumption of hydrogen accounted for ∼90 Mt/y [86]. Hydrogen is mainly used as a product for chemical and refining applications, however, hydrogen has the potential to join the energy market to become a substitute for oil products in the near future. While the global demand for hydrogen will dramatically increase by 2050 [88,89], one can hardly consider hydrogen as an efficient solution for decarbonization in the coming years primarily due to the many challenges and lack of certainty around global demand and infrastructure. Hydrogen will have to compete with batteries, electrification, and will suffer from high transportation costs and delays from the development of dedicated pipelines and distribution networks. Even if low-carbon and green hydrogen production achieves a breakeven point by 2030–2035, hydrogen will fully emerge as a vector for decarbonization only after 2040 [15,87]. In parallel, significant work will need to be realized for proving its commercial capabilities for energy applications. Nevertheless, chemical use of hydrogen and decarbonization of refining and other industrial sectors will undoubtedly be driven by the development of the hydrogen value chain.

Hydrogen is a clean energy vector that can be prepared from a variety of feedstocks, including water, natural gas, crude oil, biomass, and as a by-product from industrial processes. The carbon intensity of the various processes in the production pathway adds up to the overall carbon intensity, typically expressed in g CO_2eq_/MJ or g CO_2eq_/g H_2_ produced. The European Commission established a Certification System program called CerifHy to develop an EU-wide Guarantee of Origin scheme for low-carbon hydrogen that considers all the origins of the hydrogen and its GHG intensity. The recommended threshold for GHG intensity of low-carbon hydrogen is set at ∼60% below the intensity of hydrogen produced from natural gas by state-of-the-art steam methane reforming, currently set at 36.4 g CO_2_/MJ (5.18 g CO_2_/g H_2_) [90]. If a typical upstream natural gas would contribute approximately 10–30 g CO_2_/MJ (1.99–3.69 g CO_2_/g H_2_), this value should correspond to 70%–93% of CO_2_ capture from SMR. According to CertifHy, green hydrogen is the hydrogen from renewable energy that additionally fulfils the criteria for low-carbon hydrogen [90]. That means that in the coming years it is not the source of the feedstock for hydrogen that would matter, but its environmental impact and the type of energy used to produce it. The trend also provides new perspectives for electrified processes to produce hydrogen from methane without CO_2_ co-production. Electrified processes to produce hydrogen from methane may become particularly attractive in the future due to credits for renewable hydrogen production.

### Methane pyrolysis

**Figure 4.**
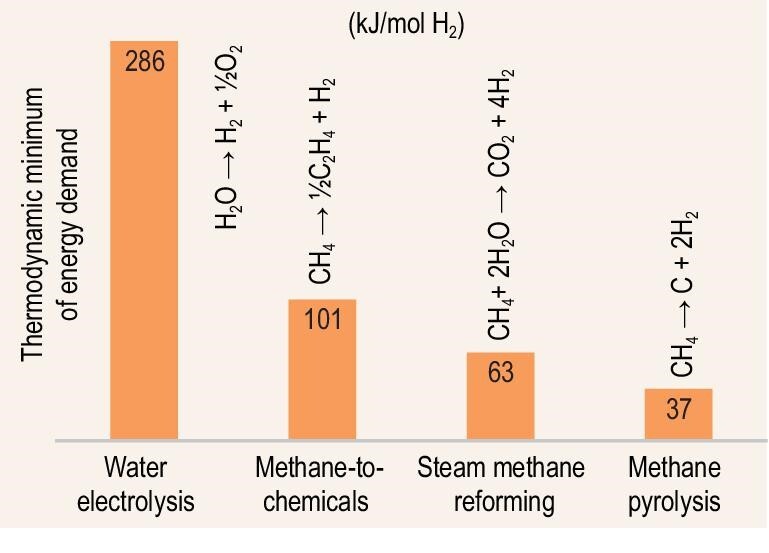
Benchmarking of the energy consumption for the production of hydrogen (kJ/mol H_2_) using different technologies: water electrolysis, methane-to-chemicals, steam methane reforming, and methane pyrolysis.

According to the International Energy Agency (IEA) [15], carbon capture, utilization, and storage (CCUS) increases the costs of hydrogen by 30%–50% from SMR, but the costs of blue hydrogen remain significantly lower than that of water electrolysis. The development of alternative electrified routes for hydrogen production from methane may bear fruit in the near future and enter into the market. Currently, one of the main CO_2_-free alternatives for blue hydrogen production is methane pyrolysis (Fig. 4). Significant attention is currently being given to this type of process based on plasma, molten salts, inductive, microwave, shock wave, and Joule-type heating to co-produce hydrogen and carbon without direct CO_2_ co-production [91]. The thermodynamic energy minimum to extract hydrogen from methane is about seven times lower in comparison to state-of-the-art water electrolysis (Fig. 4). The existing electrified semi-industrial mid-technology readiness level methods of methane pyrolysis consume between 12–16 kWh/kg H_2_ in comparison to 50–55 kWh/kg H_2_ in water electrolysis [92]. Therefore, there is a clear interest in pursuing the development of these routes.

The standard reaction enthalpy of methane conversion to solid carbon and hydrogen per mole of hydrogen is 37.4 kJ/mol H_2_. This is lower than the standard reaction enthalpy of the combined SMR and water-gas-shift (WGS) reaction, which is 41.25 kJ/mol H_2_ calculated with steam as a reactant, or 63.26 kJ/mol H_2_ for liquid water [16]. In theory, the SMR process could also be electrified, however, the reaction produces CO_2_ and requires CO_2_ purification.


\begin{eqnarray*}{\rm{Methane\ pyrolysis{:}\ C}}{{\rm{H}}}_4 \leftrightarrow {\rm{C}} + 2{{\rm{H}}}_2\end{eqnarray*}

\begin{eqnarray*}\\ {\rm{Steam\ methane\ reforming}} + {\rm{water - gas - shift{:}}}\\{{\rm{CH}}}_4 + 2{{\rm{H}}}_2{\rm{O}} \leftrightarrow {\rm{C}}{{\rm{O}}}_2 + 4{{\rm{H}}}_2\end{eqnarray*}

**Figure 5.**
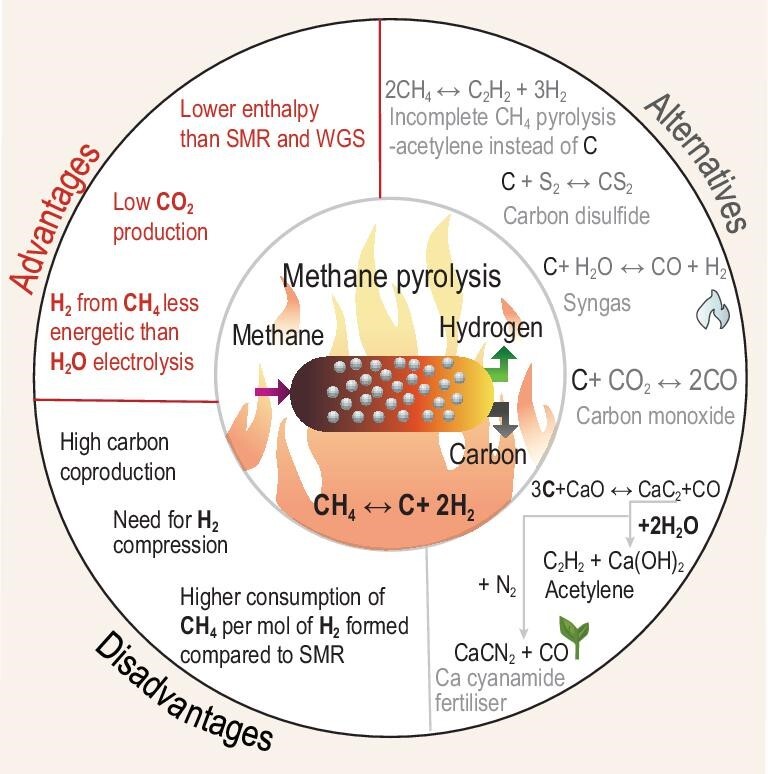
Advantages and disadvantages of methane pyrolysis and alternative valorization routes for produced carbon. Methane pyrolysis converts methane into hydrogen and carbon. Methane pyrolysis is a low CO_2_ process, and it has the advantage of being less energetic than other technologies for producing hydrogen, such as water electrolysis and combined SMR-WGS. On the other hand, some disadvantages of methane pyrolysis are the lower yield of H_2_ compared to SMR. In turn, the carbon can be valorized during the manufacture of carbon disulfide solvent, syngas, acetylene, and fertilizer.

The main disadvantages of methane pyrolysis are: (i) twice as high consumption of natural gas per ton of hydrogen in comparison to steam reforming, (ii) low pressure operation and the necessity to compress hydrogen, and (iii) significant co-production of carbon (necessary to handle solids, Fig. 5). In many cases a special reactor design is required. In this context, the valorization of carbon produced by the process is critical for the viability of the process. However, the huge difference in market sizes between hydrogen and carbon is an issue requiring serious consideration. Even if premium quality carbon could be produced by pyrolysis of methane, only a small part of this volume would be sufficient to saturate the market. For example, it is well known that methane could be transformed to a high value-added carbon black or carbon nanotubes [93], however, this process will only have a niche application due to the limits of valorization of the carbon product. It is important to consider that substitution of the hydrogen supply from SMR at a typical refinery (∼40% of the overall demand for H_2_ of a refinery) by methane pyrolysis will generate an amount of carbon several times lower than the current volume of petcoke production on the same site. As a result, within the refinery framework, the quantity of solid carbon to manage is comparable to existing operation volumes and the option to displace the H_2_ from SMR by methane pyrolysis is potentially feasible. However, in order to address the global demand for hydrogen, the development of landfilling or sequestration of the carbon product remains very challenging.

One possible solution to sequester carbon could be its utilization as a feedstock in chemical reactions with CO_2_, H_2_O, H_2_S, O_2_, or in industrial processes such as steel manufacturing or carbide production (Fig. 5). The carbon produced in pyrolysis is substantially free of contaminants. As a result of the high purity and low particle size of the carbon, applications including anthropogenic, biogenic, or native CO_2_ utilization to produce CO, carbides (CaC_2_), or new low-carbon routes for carbon gasification may be considered. The electrified gasification of the carbon ex-CH_4_ pyrolysis could be performed at substantial scales without CO_2_ formation. The latter creates opportunities for CO_2_ circularity at a refinery via hydroformylation, Fischer-Tropsch, MeOH, or other well-known routes. Metal carbide production (MgC_2_, CaC_2_) could become an interesting research avenue for carbon valorization. Those routes could facilitate the production of acetylene which could be utilized by the steel industry, for desulfurization, and for activation of nitrogen for the production of calcium cyanamide (Frank-Caro Process) [94]. Calcium cyanamide is largely used as a fertilizer and allows for the valorization of the carbon from methane in a closed loop without the release of CO_2_. Calcium cyanamide is also used as a feedstock for –N–C–N– products and could release ammonia by hydrolysis [95]. The formed calcium oxide could be recycled back into the carbide loop. This loop offers a sustainable solution for utilization of carbon from methane pyrolysis and at the same time provides an elegant way to activate nitrogen at high temperature and low partial pressure:


\begin{eqnarray*}{\rm{Ca}}{{\rm{C}}}_2 + {{\rm{N}}}_2 \to {\rm{CaC}}{{\rm{N}}}_2 + {\rm{C}}\end{eqnarray*}

\begin{eqnarray*} \\2{\rm{CaC}}{{\rm{N}}}_2 + {\rm{ }}2{{\rm{H}}}_2{\rm{O}} \to {\rm{Ca}}{\left( {{\rm{OH}}} \right)}_2 + {\rm{ Ca}}{\left( {{\rm{HC}}{{\rm{N}}}_2} \right)}_2 \end{eqnarray*}

### Non-oxidative coupling of methane (NOCM)

Considering a different perspective on the valorization of carbon in hydrogen production from methane, one can envisage leaving a part of the hydrogen together with carbon in the same molecule. This could be achieved in an electrified methane non-oxidative conversion process resulting in the co-production of valuable hydrocarbons together with hydrogen. Such hydrocarbons can be considered as a ‘carbon-sink’ for hydrogen production with a very large market size and high value which would provide significant additional economic value for the technology. For instance, the global demand for carbon black and ethylene were about 17 and 150 Mt/y, respectively [96,97]. Based on the current market size for the co-products, the world market size of carbon black will co-generate only 5 Mt/y of H_2_ from methane. At the same time, 5 Mt/y of H_2_ corresponds to the 22 Mt/y of ethylene co-production. In addition, the market size of ethylene could be significantly increased due to the potential of its utilization as a valuable platform molecule/building block to produce other types of chemicals (propylene, alpha-olefins) and fuels. In the industrialization of the co-production of ethylene with hydrogen from methane scenario, the market volume could be significantly higher. Recent technoeconomic evaluation of the industrial implantation of the NOCM shows significant potential for converting cheap natural gas to hydrogen and benzene, the prices of which were found to primarily dictate the economics of the process [98]. However, the large production of naphthalene side-product necessitates its conversion to other valuable products. This illustrates how the methane-to-chemicals process is very attractive in the current economic environment and how the hydrogen in those processes would become a co-product together with olefins and aromatics.

The NOCM process transforms methane into valuable chemicals, which plays the role of a carbon sink, and decarbonized energy vectors (such as hydrogen) while simultaneously facilitating self-sufficient H_2_ production. This would eliminate the requirement of an external hydrogen supply from water electrolysis or coal gasification. The process has been extensively studied with initial reports published at the beginning of the 1980s [99]. Since then the reaction has become an important part of the so-called ‘Methane Challenge’. The industrialization of many of the proposed routes described in the literature has been hindered by insufficient performance, severe activation conditions, challenging heat management, the narrow temperature operating window, limited conversion per pass due to thermodynamic limitations, coke formation, insufficient stability of metal-containing catalysts, and high capital intensity and energy consumption.

Challenges of heat management, a small temperature operating window, severe activation conditions, limited conversion per pass due to thermodynamic limitations, coke formation has led to several solutions being proposed based on autothermal operations like FCC with carbon rejection, heat generation materials, combination with exothermic (autothermal) reactions, and chemical looping. The main challenge lies with the high temperature requirement and fast coking which is incompatible with the reactor design in traditional refining. The thermodynamic equilibrium of the NOCM reaction and high stability of methane means the necessary temperatures to achieve conversion are above 700°C, often in the range of 800–1200°C. The unavoidable presence of unsaturated intermediates leads to coke formation and catalyst deactivation. Many options to shift the equilibrium based on membranes, hydrogen adsorbing materials, and addition of soft oxidants have been tried without significant success. However, if the reactor technology can handle a high amount of carbon formation, there is an opportunity to boost the conversion level per pass while reducing the product slate by increasing the temperature and reducing the contact time. In this context, the most promising opportunity is expected to come from the utilization of new designs of electrified reactors which are already well adapted for methane pyrolysis. The advantages of this system may arise from employing solid oxide membrane reactor technologies which remove *in situ* hydrogen while providing additional heat to the process [100,101]. Many of the electrified reactors offer a ‘cold-wall’ design with contact-free heating which avoids a significant radial gradient.

### Insufficient metal-containing material stability

Significant progress has been achieved in the synthesis of metal-containing zeolites with metal atoms located within the framework structure, such as MFI, and atomically dispersed homogeneously throughout the zeolite crystals [102]. The introduction of metal atoms heals most of the native point defects in the zeolite structure resulting in an extremely stable material. The material demonstrates superior thermal, hydrothermal (steaming), and catalytic (conversion of methane to hydrogen and higher hydrocarbons) stability, maintaining the atomically disperse metal atoms, zeolite structural integrity, and preventing the formation of silanols. These materials are also very promising as supports in many high temperature operations. Recently, significant progress has been achieved in this field with the discovery of a new insight and innovative strategy to control the defects in molecular sieves [103].

### High capital intensity and energy consumption

During the NOCM the difference in temperatures between the hottest (700–1200°C) and the coldest (−100°C) areas can be very significant and is comparable with ethane steam cracking. In addition, the methane transformation results in a low conversion per pass, the formation of significant amounts of acetylene in the C_2_ fraction, and aromatics. The catalysts producing mainly C_2_ olefins, often based on non-acidic materials containing single metal atoms, shows stable performance [104–106]. These conditions vastly complicate the recycling of the feed because of the significant energy consumption required for separation. In this context, several strategies could be adapted which include reactive separation, liquid phase acetylene hydrogenation, and membrane-type separation of methane and hydrogen. Hydrogenation of acetylene rich streams is an important part of the NOCM process development. It is important to note that the incomplete separation of CH_4_ and hydrogen will improve the performance of the NOCM conversion zone and significantly lower separation costs. The alternative option is to maximize the yield of the C_6_-C_9_ fraction downstream of the main reactor with a second catalyst. A logical solution would be to use cascade catalysis with two monofunctional zeolite catalysts working under optimized conditions. The first material will activate methane leading to a C_2_-rich effluent and the second catalyst will transform the C_2_ products to liquids at milder conditions, simplifying the back-end section. In this scenario, cryogenic separation could be completely avoided, and the process design would require liquid, solid, and gas separations only. This will decrease capital expenditure, energy consumption, and will allow a container-type unit design.

As stated above, the scope of the application of ethylene will go beyond petrochemistry. Ethylene will grow in importance as a building block to produce cleaner products. More and more technologies consider ethylene as a primary feedstock to produce goods like those currently made from oil. Ethylene could be produced from a great variety of emerging advantageous feedstocks including ethanol, methanol, ethane, and methane. The strategy to transform ethylene to valuable products is an important part of the ‘Methane Challenge’.

The specific impact of process electrification on boosting the development of methane conversion routes is still not fully understood. So-called electrified ‘cold-wall’ reactors allow many process challenges to be overcome, i.e. the management of coke formation, and results in a simpler product distribution in comparison with the many thermochemical methane conversion processes. Many options are very competitive in terms of energy efficiency when compared with the corresponding thermochemical route, but still suffer from upscaling challenges. The current industrialization of electrified methane pyrolysis processes will be extended to non-oxidative coupling processes in the upcoming years.

## CONCLUSIONS

Gas-to-chemicals offer many attractive options to multiply the effects of renewable energy on the decarbonization of the downstream industry. The direct conversion of methane to hydrogen and valuable products, such as chemicals, energy carriers and decarbonized energy vectors, will become of greater importance due to the sustainability value of decarbonizing existing assets. The former complementing the latter to produce chemicals from low-value feedstock. The development of electrified solutions to upgrade methane to chemicals with the co-production of hydrogen can help bring us closer to a paradigm shift of the sustainability challenge of future chemical production—activation of nitrogen at low partial pressure with the co-production of chemicals and clean energy vectors. If carbon from methane partially ends up in the fertilizer value chain and partially in the form of chemicals, an incredible amount of progress could be made toward achieving the net zero objective.

The sustainability value in methane conversion may provide new opportunities and concepts in chemicals and net-zero fuel production based on clean crossroad platforms: ethylene, CH_3_OH and CO/CO_2_ intermediates. CO_2_ transformation to CO with non-hydrogen routes, acetylene to ethylene hydrogenation, and hydroformylation of ethylene/olefins form the list of key priorities to address in order to meet the objectives of decarbonization. Electrified methane pyrolysis and electrified methane-to-chemicals processes yield CO_2_-free H_2_ with significantly lower electricity requirements in comparison to state-of-the-art water electrolysis. The development of new electrified processes is currently ongoing but still implies a significant risk due to the absence of convincing industrial demonstrations. Many questions remain about how the expertise in traditional thermochemical catalysis can be translated to new electrified reactor technologies. The catalytic processes in this area are still in their infancy and represent a niche research avenue.

Autothermal conversion of natural gas offers substantial potential for the decarbonization of the chemical and refining industry by avoiding GHG emissions and incorporating CO_2_ circularity within existing assets. Development of new autothermal conversion processes implies the development of robust porous materials with dual functionalities to simplify the separation load in process development. Therefore, the future development in the field of NOCM should go beyond the improvement of the individual process performance and must be assessed according to the utilization of hydrogen, heat integration, opportunistic valorization of by-products (carbon), degree of electrification, energy efficiency, and a global contribution to decrease of the overall anthropogenic carbon footprint.

It appears that there are excellent prospects to successfully address the ‘Methane Challenge’ considering the current progress in the development of cold-wall electrified reactors, bi-functional stable materials, and the economic value from decarbonization of existing assets, which methane conversion processes could benefit from. These solutions offer the potential to unlock new opportunities for the sustainable transformation of light alkanes.

## Supplementary Material

nwad116_Supplemental_File
