# Supplementary figures and images for: Methane-to-chemicals: a pathway to decarbonization
Source: Natl Sci Rev. 2023 Apr 25;10(9):nwad116. doi: 10.1093/nsr/nwad116 (PMC10411677; doi:10.1093/nsr/nwad116)

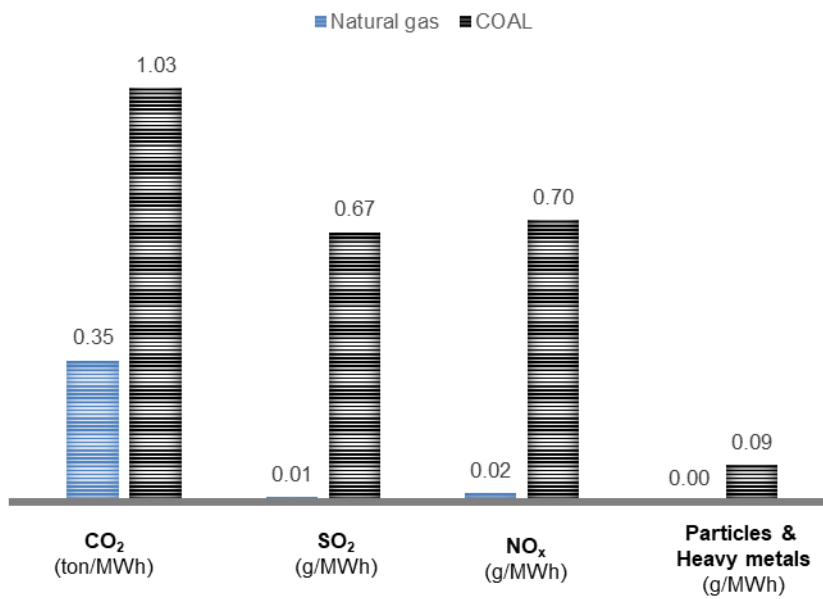

**Figure S1.** Coal (black column) vs. natural gas (blue column) emissions from 1 MWh of generated power.

Supplement: nwad116_Supplemental_File [file nwad116_supplemental_file.pdf]
